# The Effect of the combination of active vestibular interventions and occupational therapy on Balance in Children with Bilateral Spastic Cerebral Palsy: A pilot randomized Controlled trial

**Published:** 2020

**Authors:** Mehdi RASSAFIANI, Nazila AKBARFAIMI, Seyed Ali HOSSEINI, Soheila SHAHSHAHANI, Masoud KARIMLOU, Farhad TABATABAI GHOMSHEH

**Affiliations:** 1 Occupational Therapy Department, Faculty of Allied Health Sciences, Kuwait University, Kuwait. Pediatric Neurorehabilitation Research Center, Tehran, Iran.; 2 Department of Occupational Therapy, University of Social Welfare and Rehabilitation Sciences, Tehran, Iran; 3 Department of Occupational Therapy, Pediatric Neurorehabilitation Research Center, University of Social Welfare and Rehabilitation Sciences, Tehran, Iran; 4 Pediatric Neurorehabilitation Research Center, University of Social Welfare and Rehabilitation Sciences, Tehran, Iran; 5 Biostatist (ics), Social Determinants of Health Research Center University of Social Welfare and Rehabilitation Sciences, Tehran, Iran; 6 Associate Professor, Department of Ergonomics, University of Social Welfare and Rehabilitation Sciences, Tehran, Iran

**Keywords:** Cerebral Palsy, Vestibular Intervention, Balance, Activity of Daily Living, Occupational Therapy

## Abstract

**Objective:**

The current study aimed to examine the effect of the combined administration of active vestibular interventions and occupational therapy on balance and the relationship between balance changes and Activity of Daily Living in school-aged children with cerebral palsy (CP).

**Materials & Methods:**

Twenty-four children with Spastic CP, at the level I and II (according to the “Gross Motor Function Classification System) aged 7-12 years were enrolled and randomly assigned into control and intervention groups. Pediatric Balance Scales and Bruininks-Oseretsky Test of Motor Proficiency II were employed to assess the functional balance changes as well as Force Plate (eyes closed and open) to assess changes in the parameters of balance (e.g. center of pressure excursion). The activity of Daily Living was assessed by “Activity Scales for Kids (performance version)”. Participants in the intervention group received active vestibular intervention for 20 min and a regular occupational therapy program for 25 min. The control group received a regular occupational therapy program for 45 min. Interventions were provided 3 d/week for 6 weeks in each group. The participants were assessed in three stages: baseline, immediately after, and eight weeks after the intervention. Data were analyzed by ANOVA and linear regression.

**Results:**

The results demonstrated that only functional balance, according to Pediatric Balance Scales scores, was significantly increased in the active vestibular interventions group (p=0.02). There was no significant association between functional balance and Activity of Daily Living (P>0.05).

**Conclusion:**

The combined administration of active vestibular interventions and occupational therapy could improve the functional balance in children with spastic CP. It may be related to the reorganization of the vestibular system with a controlled and precise application of stimuli.

## Introduction

Cerebral Palsy (CP) is a permanent and non-progressive childhood syndrome that negatively affects brain development and results in movement and postural disorders that severely limits the activity (1). Several factors affect the balance in children with CP, including body alignment, muscle and postural tone, and movement strategies that control spontaneous sway and recovery from perturbations to stance stability. The sway amplitude is larger and more irregular in children with CP compared to their healthy counterparts (2). Children with CP have remarkable problems in adapting the amplitude of postural responses to perturbations of increasing distance and velocity of the center of mass or pressure (COM or COP) (2, 3). For postural control, there should be a balance between the complex interactions of a person with the environment and the tasks. It involves controlling the body’s position in space for the dual purpose of balance and orientation. Balance involves a complex interaction between musculoskeletal and neural systems. The vestibular system plays a viable role in postural control (balance abilities) through sensing and perceiving self-motion, vertical orientation, and controlling movement of COM or COP, and stabilizes the head during postural movements (2). The vestibular system, as compared to other sensory systems, may be an important modality that can preserve conscious changes in balance strategy, as well as initiate and maintain the compensatory mechanism in shorter response time to balance perturbation (4).

The vestibular system is a practical modality and powerful input for therapeutic interventions (5). Vestibular intervention (i.e., active vestibular stimulation and passive vestibular stimulation) along with adequate intensity and timing can effectively influence the reorganization and facilitate neural and synaptic plasticity in the vestibular pathways (6). The positive effects of the vestibular intervention on muscle tone and gross motor function are also demonstrated in children with CP (6, 7); however, there is a dearth of evidence regarding the effectiveness of vestibular interventions, in both active and passive forms, on balance. 

Active vestibular interventions generate through active and volitional head only or whole-body rotation in the space; and passive vestibular interventions refer to the stimulation in which the subjects play no active role in producing the head or body rotation (8, 9). Active vestibular interventions are performed in the context of meaningful, purposeful, and goal-directed activities through sensory feedback derived from the production of adaptive behaviors. Thus, children can meet the “just right” challenge (refers to situations that therapists provide challenges intended to empower children and also to show them that they are able to improve) and learn new movement patterns (10). Active interventions, as compared to passive interventions, appear to be more attractive in children as they can control themselves and predict the movements (10). Therefore, the main aim of this study was to examine the effect of the combination of the active vestibular interventions and occupational therapy on balance, as well as on the Activity of Daily Living (ADL) of school-aged children with bilateral spastic CP in areas of self-care and dressing.

In this study, it is hypothesized that a combination of active vestibular interventions and occupational therapies can influence the children’s balance. Moreover, these changes would be increased immediately after six weeks and would be maintained for two months after completing the intervention. Also, according to the ‘international classification of function, disability, and health’ (ICF), function of children with CP is influenced by impairment or limitation in one or more of the body function and structure, activity and participation, so we hypothesized that improving the children’s balance would increase their independence in ADL (11).

## Materials & Methods

This is a pilot Randomized Controlled Trial, in which one of its arms was blinded (evaluators). 

Subjects

**Figure 1:**
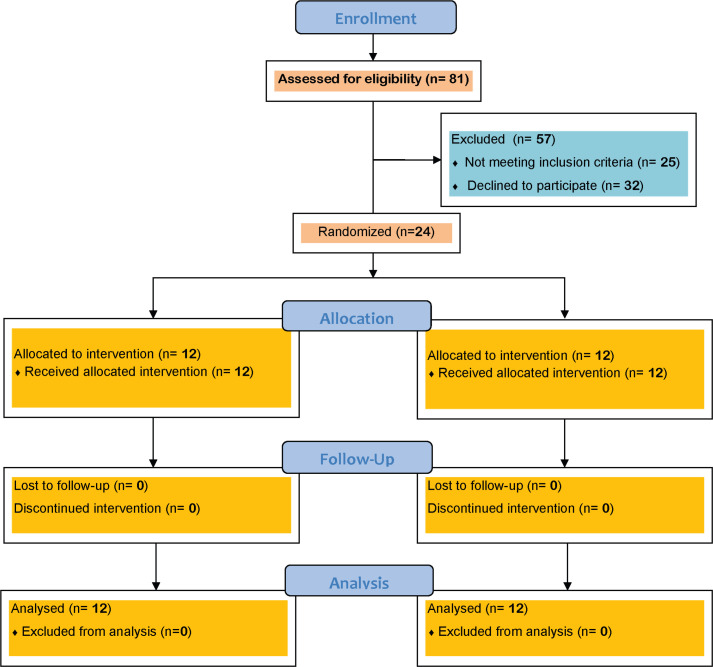
The flowchart for the study design and participant selection

In total, 24 children (based on equation1) with bilateral spastic CP participated in this study(10 boys and two girls, average age 8.41±1 years in the intervention group, and seven boys and five girls, with average age 9.18±1 years in the control group) (Figure 1) and were randomly (via envelope; envelope titled number1 assigned in control group and envelope titled number2 assigned in intervention group) assigned into the intervention and control groups. The subjects were recruited from four pediatrics rehabilitation centers. Inclusion criteria were being diagnosed by a neurologist; aged 7-12 years; having a CP of level I or II according to the Gross Motor Function Classification System (GMFCS); being able to stand independently without any support for at least 30 seconds (12); no history of surgical interventions or injection, Botulinum Toxin injection and surgery, six months prior to the study as well as not being a candidate for toxic injection and surgery; having a score between 1 to 3 according to Modified Ashworth Scales (MAS) in both hip and plantar flexors, and understanding verbal comments. The exclusion criteria were vision or hearing impairments and having uncontrolled epilepsy. The current study is approved by the University of social welfare and rehabilitation sciences (USWR) Ethical committee. Besides, written informed consent was obtained from the parents of all participants.

Equation 1: Sample size estimation for this study

n= )_1_^2^+ 𝛿_2_^2^ ((Z_1-__𝛼__/2 _+Z_1-__β_ )^2^**/**𝛥^2^

𝛿_1=variance of baseline values within a treatment group_

𝛿_2= variance of baseline values within a control group _

𝛥=_difference between the sample means_

2.2: Measurements

2.2.1: Balance parameters

The balance was evaluated by analyzing the time-varying coordinates of the COP. The COP signals were collected at a sampling frequency of 100 HZ, over a period of 20 seconds using a single piezoelectric force platform (model 9286; Kisler, Switzerland, Bioware 4-0-12). All participants were tested in the standing position with both eyes opened and closed (three times). The participants were asked to stand independently, with bare feet, on the markers in the center of the platform with arms at the sides, while looking straight to the spot in front of them. At first, two conditions were conducted, both with open and closed eyes. Meanwhile, they could rest during the test on a chair for at least five minutes. The mean distance (MDIST) and mean velocity (MVEL) of COP were computed with each A-P and M-L direction along the x-axis and y-axis according to the Prieto formula (13). The mean values obtained after three times of testing was calculated for each participant. The higher the score, the better the balance ability. Data filtering and COP calculations were performed using the MATLAB software (R2010a). For data filtering, the AP and ML time series were passed through a fourth-order zero-phase Butterworth low-pass digital filter with a 5-Hz cut-off frequency (13). 

Functional balance

The functional balance was tested using the PBS and the balance subtest of BOT-2. PBS comprises 14 items with five ordinary scales options ranging from 0 to 4. Its maximum score is 56, which indicates the highest possible ability (14). The test-retest reliability (intraclass correlation coefficient (ICC) >0.95) and inter-rater reliability (ICC = 0.98-1.00) of PBS for CP children were reported as excellent (14). BOT-2 consists of eight subtests: six for the balance skills and three for dynamics, with ordinary scales. The maximum overall score is 36, which indicates the best balance skill (15). The Inter-rater reliability of the balance subtest was found to be high (r = 0.99). Test-retest reliability, however, was different (15).

Other clinical measurements

The gross motor function was assessed by GMFCS (1). The GMFCS scales contain five distinct motor levels, which children in level I can walk without limitation, and in level V use the wheelchair for transportation (16). Spasticity was assessed using MAS, which ranges from 0 (indicating normal tonus) to 4 (i.e., rigidity), based on the degree of muscle resistance against the passive movement (17). 

Changes in functional activities were assessed using the “Activity Scales for Kids performance version” (ASK). The ASK includes 30 items with seven sub-domains (i.e., personal care, dressing, other skills, locomotion, play, standing skills, transfers) that each has five ordinal scales ranging from 0 to 4. The higher the score, the higher the functional abilities (18).  The Persian version of ASK has an acceptable content validity (0.79 for university professors and 0.86 for parents) and reliability rates (Internal consistency= 0.997 and Test-retest reliability ;ICC = 0.998)(19).

All measurements were performed for three times (before intervention (baseline), immediately after the intervention (after), and eight weeks after completion of the intervention (follow-up)) by two occupational therapists that were blinded to the grouping of the participants (one for laboratory measurements and one for functional and clinical assessments). 

Intervention

In the active vestibular interventions group, participants received regular occupational therapy for 25 minutes and active vestibular stimulation for 20 minutes. In the control group, the participant received only regular occupational therapy based on neurodevelopmental treatment (NDT) (i.e., active and passive stretching on spastic muscles and facilitation technique to improve balance reactions as encourage children to walk on balance beam or ladder) for 45 minutes (20). Interventions were performed for 15 sessions in six weeks.

The active vestibular intervention was performed according to the protocol developed by Hosseini (21) using the platform swing, bolster swing, net swing, CP ball, tilt board, trampoline, and ramp. The room setting was arranged to engage the children, create play context, ensuring physical safety, offering suitable stimulation, and providing the just-right challenge. The first session was intended to establish a close relationship with children and familiarizing them with the vestibular equipment. They were free to use the vestibular equipment with their pleasant position (e.g., lying, sitting, and kneeling). 

In the subsequent sessions, the therapist guided the children to perform various types and intensity of stimulation that would result in new adaptive balance responses. For example, in the beginning, vestibular stimulation offered very slow and linear movements at lying position with support. Then, it gradually progressed to more difficult situations with faster and more angular movements at the standing position with minimal support. The therapist noticed that the children must receive the just-right challenge with successful performance. The interventions were implemented by four occupational therapists that were trained through two sessions and were blinded to the grouping.

2.4: Data analysis and statistical methods:

Kolmogorov-Smirnov (K.S) and chi-square tests were applied to evaluate the normal distribution for numerical and dichotomous variables, respectively. Repeated measured ANOVA was used to compare the force plate parameters for 2 conditions (i.e., eyes open and closed) x 2 (group) x 3 (time). Repeated measured ANOVA was also used to analyze ASK, PBS, and BOT 2 results at the 2 (group) x 3 (time). Linear regression was used to analyze the association between the variables (ASK with the force plate parameters, PBS, and BOT 2 scores separately). The Alpha level for the ANOVA's and correlation coefficients was set at 0.05. Data analysis was performed using SPSS version 17. 

## Results

Out of 81 children with CP (Figure 1), 24 were able to participate in the current study and were randomly divided into intervention and control groups (10 boys and two girls, with a mean age of 8.41 years (SD=1.00) in the active vestibular intervention, and seven boys and five girls with a mean age of 9.18 years (SD=1.65) in the control group) (Figure 1). The majority of participants (83.33%, N=20) had a brain CT or MRI before participating in this study with periventricular leukomalacia. Further demographic characteristics are summarized in Table 1. All participants in both groups completed the intervention sessions.

According to the results, balance parameters were improved after providing the intervention: MVELAP and MVELML under both open eyes and closed eyes conditions scores across time in each group. There was neither a significant main effect of group nor a significant effect of time (between two groups) on all the variables (p>0.05) (Table 2).

The results of the ANOVA indicated that balance scores were improved after providing the intervention ( PBS, BOT-2). Also ASK scores were enhanced in each group (Table 3). There was neither a significant main effect of group nor a significant effect of time (between two groups) on all the variables, except for PBS scales (F_ (1.22)_=6.84, P=0.02), indicating that, over time, only changes in PBS were dependent on the type of the active vestibular intervention group (Table 3).

The Mean and Standard Deviation (mean±SD) of MVELAP and MVELML scores and PBS scales of each group at various measurement times (i.e., baseline, after the intervention, and Follow-up) are described in Table 4.

The results of correlation analysis using mean differences between baseline and follow-up tests (Table 5) indicated that all changes in balance parameters were not significantly correlated with changes in ASK scores (p<0.05) and, therefore, were not considered for further analysis.

**Table 1:** Demographic characteristics of two study groups

**Characteristics**	**Control (N=12)** **N (%)**	**Intervention (N=12)** **N(%)**	**P **_value_
**Age in year (M±SD) **	8.42±1.00	9.19±1.66	0.18
**BMI (M±SD)**	17.99±5.53 kg/cm^2^	20.94±4.74 kg/cm^2^	0.17
**Gender:**			
Boy Girl	10 (83.3) 2 (16.7)	7 (58.3) 5 (41.7)	0.82
**Type of cerebral palsy:**			
Quadriplegia Diplegia	8 (66.7) 4 (33.3)	7 (58.3) 5 (41.7)	0.87
**GMFCS:**			
I II	2 (16.7) 10 (83.3)	5 (41.7) 7 (58.3)	0.84
**Ashworth (ankle):**			
2 3	5 (41.7) 7 (58.3)	5 (41.7) 7 (58.3)	0.97
**Ashworth (hip):**			
1 2 3	1 (8.3) 7 (58.3) 4 (33.3)	2 (16.7) 8 (66.6) 2 (16.7)	0.99

**Table 2:** Results of repeated measure ANOVA: at the 2 (Group: Control, Intervention) x 3 (Time: baseline, After intervention; and Follow-up) x2(open eyes, closed eyes).

**Measurement**		**Analysis**								
		Factor(time)			Interaction (group*factor)			Between group		
		F^a^	P	Effect Size (η ^2 ^)	F^a ^	P	Effect Size (η^ 2 ^)	F^a^	P	Effect Size (η ^2 ^)
**MDISTAP (mm):**	Open eyes	0.01	0.921	<0.001	0.48	0.49	0.02	0.71	0.41	0.03
	Closed eyes	1.84	0.19	0.08	4.34	0.05	0.17	0.55	0.47	0.02
**MDIST ML (mm):**	Open eyes	0.19	0.66	0.01	0.03	0.87	0.001	0.04	0.85	<0.001
	Closed eyes	0.42	0.52	0.02	1.79	0.19	0.07	0.03	0.87	<0.001
**MVELAP (mm/s):**	Open eyes	15.40	0.001*	0.41	0.11	0.74	0.01	0.51	0.48	0.02
	Closed eyes	16.58	0.001*	0.43	0.17	0.67	0.01	1.88	0.18	0.08
**MVELML (mm/s):**	Open eyes	14.47	0.001*	0.39	0.16	0.69	0.01	1.88	0.18	0.08
	Closed eyes	6.72	0.003*	0.23	0.66	0.52	0.03	2.98	0.09	0.12

Notes: Mean Distance in Anterior- Posterior direction (MDISTML), Mean Distance in Mediolatral direction (MDISTML), Mean Velocity of COP in Anterior- Posterior direction (MVELAP), Mean Velocity of COP in Mediolateral direction (MVELML), “Pediatric Balance Scales” (PBS), the balance subtest of Bruininks-Oseretsky Test of motor proficiency Instruments (BOT-2), “Activity Scales for Kids performance version” (ASK) , ^a^ df=1 and 22 , *Statistically significant.

**Table 3:** Results of repeated measure ANOVA: at the 2 (Group: Control, Intervention) x 3 (Time: baseline, After intervention; and Follow-up)

**Measurement**		**Analysis**								
		Factor(time)			Interaction (group*factor)			Between group		
		F^a^	P	Effect Size (η ^2 ^)	F^a ^	P	Effect Size (η^ 2 ^)	F^a^	P	Effect Size (η ^2 ^)
**PBS**		18.69	<0.001*	0.46	12.06	0.002*	0.35	6.84	0.02*	0.24
**BOT2**		52.09	<0.001	0.70	2.51	0.127	0.10	0.03	0.85	<0.001
**ASK**		23.32	<0.001	0.52	7.56	0.012	0.26	0.30	0.59	0.01

Notes: “Pediatric Balance Scales” (PBS), the balance subtest of Bruininks-Oseretsky Test of motor proficiency Instruments (BOT-2), “Activity Scales for Kids performance version” (ASK) , ^a^ df=1 and 22 , *Statistically significant.

**Table 4:** Mean and Standard Deviation (mean±SD) in each group (Control, Intervention), at each time (baseline, After intervention, and Follow-up)

**Measurement**			**First time (** **Before)**		**Second time (After)**		**last** **time** **(Follow up)**		P_value (_main effect for time within each group)
		Groups	Mean	SD	Mean	SD	Mean	SD	
**MVELAP (mm/s):**	**Open eyes**	Control (N=12)	70.56	16.23	66.16	4.81	54.11	15.19	.008
		Intervention (N=12)	67.15	23.68	69.15	4.61	53.30	16.45	.036
		P_value(_ main effect for time betwen each group)	.685		.257		.902		-
	**Closed eyes**	Control (N=12)	67.29	18.65	61.13	26.25	50.92	14.04	.016
		Intervention (N=12)	59.21	23.38	48.62	14.76	45.92	12.08	.012
		P_value(_ main effect for time betwen each group)	.360		.164		.360		-
**MVELML (mm/s):**	**Open eyes**	Control (N=12)	78.51	16.66	74.32	18.39	67.699	19.839	.056
		Intervention (N=12)	73.12	16.37	63.16	17.72	59.79	13.88	.005
		P_value(_ main effect for time betwen each group)	.432		.143		.270		-
	**Closed eyes**	Control (N=12)	80.10	22.39	71.58	22.01	76.05	15.47	.336
		Intervention (N=12)	72.54	15.294	58.75	14.94	61.71	19.92	.037
		P_value(_ main effect for time betwen each group)	.344		.109		.062		-
PBS:		Control (N=12)	27.17	5.71	28.33	4.91	28.197	5.29	.256
		Intervention (N=12)	27.92	7.63	36.33	6.789	37.089	6.492	.002
		P_value(_ main effect for time betwen each group)	.788		.003		.001		-

Notes: Mean Velocity of COP in Anterior- Posterior direction (MVELAP), Mean Velocity of COP in Mediolateral direction (MVELML), “Pediatric Berg Balance Scales” (PBS)

**Table 5:** Correlations of all calculated force plate variables, BOT2 and PBS scores with ASK scores on the bases of mean differences between baseline and follow-up test

		**ASK**	
**Measurement**		**r**	**p**
MDISTAP (mm):	Open eyes	-0.06	0.79
	Closed eyes	0.33	0.12
MDIST ML (mm):	Open eyes	-0.01	0.95
	Closed eyes	0.12	0.56
MVELAP (mm/s):	Open eyes	-0.25	0.26
	Closed eyes	0.17	0.42
MVELML (mm/s):	Open eyes	0.17	0.43
	Closed eyes	0.04	0.86
PBS		0.32	0.13
BOT2		0.03	0.89

Notes: Pearson’s correlation co efficient (r) and two tailed significant (p). Mean Distance in Anterior- Posterior direction (MDISTML), Mean Distance in Mediolatral direction (MDISTML), Mean Velocity of COP in Anterior- Posterior direction (MVELAP), Mean Velocity of COP in Mediolatral direction(MVELML), “Pediatric Balance Scales” (PBS), the balance subtest of Bruininks-Oseretsky Test of motor proficiency Instruments (BOT-2), “Activity Scales for Kids performance version” (ASK).

## Discussion

In this study, it was hypothesized that the combined provision of active vestibular intervention and occupational therapy would improve both balance parameters and functional tests of balance. It was found that the combination of active vestibular intervention and occupational therapy may improve only functional balance (according to PBS) in children with CP. Which indicates that this improvement had effects on functional activities that was quantified by ASK, but did not appear to affect activity level according to ICF. 

The results did not show any significant difference between the two groups concerning the balance parameters but revealed remarkable changes in MVELAP and MVELML parameters in the active vestibular intervention group. The vestibular system, as a sensory system, plays a major role in resolving sensory conflicts and sensory reweighting in static postural control (22). In the current study, it was suggested that the active vestibular stimulation may reorganize the vestibular system, thereby resulting in the activation of vestibular afferents. These changes could compensate for the deficits of the other sensory systems and provide appropriate sensory information for the “central nervous system” to align the body to vertical orientation in the environment (4). This process can result in the selection of proper postural strategies, which in turn produces appropriate postural responses (22). It has been reported that changes in velocity parameters of balance, particularly in the ML direction, in healthy adults is related to the hip strategy (23). Furthermore, the vestibular information do not play an essential role in the initiation or execution of normal ankle strategy in that somatosensory information is sufficient to produce normal ankle strategy responses (22). Children with CP can maintain balance and compensate for muscle weakness by preferring one strategy to another or adopting a unique biomechanical postural alignment (23). The results of this study showed improvement in MVELAP and MVELML parameters in both intervention and control groups, although no difference was found between the intervention and control groups. This indicates that both types of interventions may result in the facilitation of vestibulospinal reflex.

As mentioned earlier, little evidence is available on interventions related to vestibular stimulation for training or improving balance abilities in children with spastic CP. The hippo therapy and horseback riding, as interventions for improving the balance, posture, and walking abilities, have physiological mechanisms such as vestibular stimulation (24). The rhythmical and reciprocal horse movements in repetitive patterns move the rider's head and body in such a manner that perturbs the otolith organs (24, 25). Although we could not find a study comparing these two interventions, we can compare these results with those used in hippotherapy (which is different in some aspects such as horse temperature and extent of required balance). Few studies (with pre-post test methodology) have reported improvements in balance parameters and PBS scores after the hippotherapy for over 8 weeks in children with spastic CP. They showed that these changes may be associated with variations of the velocity, direction, and stride of horse movements, which result in normalization of tone, recruitment of hip strategy, improvement in co-ordination, and postural and equilibrium responses, which help to learn the postural regulation patterns (26). The present study demonstrated similar results in PBS within a short period (6 weeks) by maintaining the effects 8 weeks later. This improvement may be attributed to children's engagement in a playful and joyful environment and application of a just-right challenge. 

Moreover, the children were able to regulate the intensity and type of balance tasks, attempt to maintain their body alignment in the midline, learn the balance strategies during each task, and to generalize these experiments to the other balance situations. 

There was no statistically significant difference between both interventions concerning the balance parameters and BOT-2 scores, which may be due to the low sensitivity of the measures to detect small changes in children with CP or short duration of the intervention.

Most previous studies suffer from some methodological weaknesses, such as not using suitable measures and tools, intervention duration as well as the absence of a control group and follow-up reports. Therefore, in the current study, these weaknesses were considered, and suitable measures and tools were selected. Based on the fact that minor changes in children’s abilities can cause major functional or physiological improvements in children with CP, the available measures (i.e., MAS, ASK, PBS, and BOT-2) may detect the presence or absence of a skill and may not be sensitive to changes in gradation of ability within a skill. Moreover, the growth, development, and motor learning skills of children with CP are very slow and, hence, the duration of interventions is very important (27, 28). Therefore, it’s recommended that the duration of interventions be longer than 6 weeks with more sensitive tests to show changes in parameters. Another limitation of the current study is not blinding the participants, which may have influenced the outcomes, such as ASK, and it is suggested that this issue be considered in future studies. 

The number of the participants of this study was low and, similar to other previous studies, mainly due to the budget limitations and willingness of children and families to attend in a study with long term interventions and assessment. Which reduced the power of the study and imposed limitations in generalizing the results. Therefore, it would be useful to increase the number of participants in future studies. 

## In Conclusion,

According to the results, the combined use of active vestibular intervention and occupational therapy led to better balance than the sole administration of occupational therapy in children with CP. This improvement is quite limited, as only the PBS increased significantly more in the active vestibular intervention group. Furthermore, the intervention resulted in a higher increase in the “ASK” score in the control group. However, the improvement in the “ASK” score was not directly/linearly related to the improvements in the balance. We cannot claim the sole influence of the active vestibular intervention on the children’s performance. Therefore, it is recommended that future research not only investigate the effect of vestibular intervention alone on the children’s function but also compare the differences between active and passive vestibular intervention in these children with a larger sample size. 
